# Impact of intrapartum antimicrobial prophylaxis upon the intestinal microbiota and the prevalence of antibiotic resistance genes in vaginally delivered full-term neonates

**DOI:** 10.1186/s40168-017-0313-3

**Published:** 2017-08-08

**Authors:** Alicja Nogacka, Nuria Salazar, Marta Suárez, Christian Milani, Silvia Arboleya, Gonzalo Solís, Nuria Fernández, Lidia Alaez, Ana M. Hernández-Barranco, Clara G. de los Reyes-Gavilán, Marco Ventura, Miguel Gueimonde

**Affiliations:** 1 Department of Microbiology and Biochemistry of Dairy Products, Instituto de Productos Lácteos de Asturias. Consejo Superior de Investigaciones Científicas (IPLA-CSIC), Ctra. Infiesto s/n, 33300 Villaviciosa, Asturias Spain; 2 Pediatrics Service, Hospital Universitario Central de Asturias, SESPA, Oviedo, Asturias Spain; 3 Laboratory of Probiogenomics, Department of Life Sciences, University of Parma, Parma, Italy; 4 Current address: APC Microbiome Institute, University College Cork. Cork, Ireland & Teagasc Food Research Centre, Moorepark, Fermoy, Cork, Ireland

**Keywords:** Intestinal microbiota, Microbiome, Antibiotics, Neonate, Intrapartum antimicrobial prophylaxis, Antibiotics

## Abstract

**Background:**

Disturbances in the early establishment of the intestinal microbiota may produce important implications for the infant’s health and for the risk of disease later on. Different perinatal conditions may be affecting the development of the gut microbiota. Some of them, such as delivery mode or feeding habits, have been extensively assessed whereas others remain to be studied, being critical to identify their impact on the microbiota and, if any, to minimize it. Antibiotics are among the drugs most frequently used in early life, the use of intrapartum antimicrobial prophylaxis (IAP), present in over 30% of deliveries, being the most frequent source of exposure. However, our knowledge on the effects of IAP on the microbiota establishment is still limited. The aim of the present work was to evaluate the impact of IAP investigating a cohort of 40 full-term vaginally delivered infants born after an uncomplicated pregnancy, 18 of which were born from mothers receiving IAP.

**Results:**

Fecal samples were collected at 2, 10, 30, and 90 days of age. We analyzed the composition of the fecal microbiota during the first 3 months of life by 16S rRNA gene sequencing and quantified fecal short chain fatty acids by gas chromatography. The presence of genes for resistance to antibiotics was determined by PCR in the samples from 1-month-old infants. Our results showed an altered pattern of intestinal microbiota establishment in IAP infants during the first weeks of life, with lower relative proportions of *Actinobacteria* and *Bacteroidetes* and increased of *Preoteobacteria* and *Firmicutes*. A delay in the increase on the levels of acetate was observed in IAP infants. The analyses of specific antibiotic resistance genes showed a higher occurrence of some β-lactamase coding genes in infants whose mothers received IAP.

**Conclusions:**

Our results indicate an effect of IAP on the establishing early microbiota during the first months of life, which represent a key moment for the development of the microbiota-induced host homeostasis. Understanding the impact of IAP in the gut microbiota development is essential for developing treatments to minimize it, favoring a proper gut microbiota development in IAP-exposed neonates.

**Electronic supplementary material:**

The online version of this article (doi:10.1186/s40168-017-0313-3) contains supplementary material, which is available to authorized users.

## Background

The human gut harbors a rich and complex microbiota whose establishment begins in early life. This microbiota contributes to an appropriate gut development and intestinal barrier function, resulting essential for the metabolic and immune homeostasis of the host [1, 2]. During the last years, an increasing amount of scientific evidence has underlined the important role of the early-life gut microbiota establishment and the development of such microbiota, in the later health of the individual [2–6]. This early microbiota-host interaction affects not just the local gut environment but also distal organs [7, 8]. Therefore, any disturbance on the intestinal microbiota development process in these early moments may have important implications for the infant’s health and also for the risk of disease later on life. Indeed, an aberrant early microbiota has been found to precede the development of diseases [9–11].

Traditionally, infants have been considered sterile in utero, but recent data support the occurrence of a certain degree of microbial exposure prior to delivery [12]. At birth, the microbial exposure becomes massive and the newborn is rapidly and densely populated by a complex microbiota. This microbial colonization begins with facultative anaerobic and aerotolerant microorganisms which reduce the intestinal environment allowing the further establishment of strict anaerobes, such as *Bifidobacterium* and *Bacteroides* [13, 14]. After the initial colonization steps, the infant microbiota often appears dominated by *Actinobacteria*, frequently accompanied also by high levels of *Proteobacteria* [15–17]. This intestinal colonization process is going to be dependent on both genetic and environmental factors [18]. Among the last ones, many different prenatal and postnatal conditions may be affecting the colonization process, including gestational age at birth, mode of delivery, use of antibiotics, or feeding habits [17–21]. Some of these factors, such as being born by C-section or receiving antibiotics, have been associated with an increase in the risk of later disease [22].

Antibiotics are among the drugs most frequently used in early life. During the last years, different animal studies have demonstrated that reduced exposure to microbes early in life is associated with an increased risk of later disease [11, 23]. Moreover, antibiotics-induced microbiota dysbiosis in early life leads to increased susceptibility to allergic and metabolic disorders [24–26]. The most frequent cause of exposure to antibiotics during the perinatal period is the use of intrapartum antimicrobial prophylaxis (IAP), which is present in over 30% of deliveries [27]. Several studies have shown that early postnatal antibiotic exposure disturbs the natural establishment of the intestinal microbiota in the newborn with potential negative influence in later health [28, 29]. However, with the exception of some preliminary culture-based assessment [30], only recently studies focusing on the impact of IAP on the microbiota development, in both preterm and full-term newborns, have started to be available [17, 31–33]. In spite of this, our knowledge on the effects of IAP on the intestinal microbiota development is still limited.

The aim of the present study was to evaluate the impact of IAP on the establishment of the gut microbiota in the newborn. To avoid the presence of potential confounders, we focused on vaginally delivered full-term infants remaining healthy during the length of the study. To this end, we used 16S rRNA gene sequence-based fecal microbiota analysis, gas chromatography (GC) for quantification of fecal short chain fatty acids (SCFAs) as a measure of microbiota metabolic activity, and PCR for the detection of specific antibiotic resistance genes.

## Methods

### Study participants

The study was approved by the Regional Ethical Committee of Asturias Public Health Service (SESPA), and an informed written consent was obtained from each infant’s parents. The study included 40 full-term (gestational ages >37 weeks) vaginally delivered infants born after an uncomplicated pregnancy at the Central University Hospital of Asturias (Northern Spain). Eighteen of the mothers received intrapartum antimicrobial prophylaxis (in all cases, an initial dose of 5 million units of penicillin followed by 2.5 million units every 4 h until delivery; in most cases, the mothers received three or less doses), due to confirmed or suspected vaginal colonization by group-B-streptococci (IAP group); whilst, the other 22 mothers, all of them being culture negative for group-B-streptococci, were not exposed to antibiotics (no-IAP group). None of the mothers received antibiotics during pregnancy or the postnatal period, other than the above mentioned IAP, and none of the infants received antibiotics during the duration of the study. Eighteen infants from the no-IAP and 11 from the IAP group were exclusively breast-fed during the study period whilst the remaining newborns (four in the no-IAP and seven in IAP group; difference no statistically significant) received infant formula milk. All infants were discharged from the hospital after 2 or 3 days of life.

### Sample collection

Fecal samples were collected at 2, 10, 30, and 90 days of age. A fresh fecal sample was taken in a sterile container by the parents and immediately frozen at −20 °C. Samples were sent within 1 week to the laboratory where they were stored at −80 °C until DNA extraction.

### Fecal DNA extraction

Fecal samples were thawed, weighed, diluted 1/10 in sterile PBS solution, homogenized in a LabBlender 400 stomacher for 5 min, and DNA was extracted using the QIAamp DNA stool kit (Qiagen GmbH, Hilden, Germany) as previously described [14]. Extracted DNA was kept frozen at −80 °C until analysis.

### 16S rRNA gene sequence-based microbiota analysis

Partial 16S rRNA gene sequences were amplified from extracted DNA using primer pair Probio_Uni and /Probio_Rev, which target the V3 region of the 16S rRNA gene sequence [34]. 16S rRNA gene sequencing was performed using a MiSeq (Illumina) according to the protocol previously reported [34]. Following sequencing, the obtained individual sequence reads were filtered by the Illumina software to remove low quality sequences. All Illumina quality-approved, trimmed, and filtered data were exported as .fastq files. The .fastq files were processed using a custom bash script based on the QIIME software suite [35]. This script performs a quality control of the sequences and retains only those with a length between 140 and 400 bp as well as mean sequence quality score >20, truncates sequences at the first base if a low quality rolling 10 bp window was found, and removes reads with homopolymers >7 bp as well as with mismatched primers. The custom bash script then integrates Qiime python scripts in order to generate de novo 16S rRNA Operational Taxonomic Units (OTUs) with ≥97% identity using uclust [36], remove OTUs with less than 10 sequences, pick a random reference sequence for each OTUs, classify OTUs down to the genus level by means of the SILVA database v.123 [37], remove chimeric sequences using ChimeraSlayer (http://microbiomeutil.sourceforge.net/), and calculate alpha-diversity rarefaction curves using chao1, Shannon, and observed number of OTUs indices.

### SCFAs analysis

The concentration of SCFAs in feces (mM) was determined in a chromatographic system composed of two 6890 N GC (Agilent Technologies Inc., Palo Alto, CA, USA) connected to a FID and a MS 5973N detector as previously described [38].

### Detection of antibiotic resistance genes in fecal samples

The occurrence of different antibiotic resistance genes in fecal samples from 1-month-old infants was determined by PCR (UnoCycler, VWR International, Pennsylvania, USA) using previously described primers (Table 1). All oligonucleotides were purchased from Macrogen Europe (Macrogen, Amsterdam, The Netherlands). PCRs were performed in a total volume of 25 μl containing 1 μl of fecal DNA extract as a template. The reaction mixture was composed of 1× Dream PCR Master Mix (Thermo Fischer Scientific, San Jose, CA, USA) using 0.2 μM of each primer. The thermal cycle program consisted of the following time and temperature profile: an initial cycle of 94 °C for 5 min, 35 cycles of 30 s at 94 °C, 30 s at the annealing temperature of the corresponding primer pair (Table 1) and 1 min at 72 °C, and a final extension step of 7 min at 72 °C. Amplified products were subjected to gel electrophoresis in 1% agarose gels and were visualized by ethidium bromide staining.

**Table 1:** Primers and annealing temperatures used for the detection of antibiotic resistance genes by PCR

Gene	Oligonucleotide	Annealing T°	Antibiotic group	Resistance mechanisms	Ref.
*tet*(W)	F-AAGCGGCAGTCACTTCCTTCCR-TCAAGTATCCCAGCGAAACC	60	Tetracyclines	Ribosomal Protection Protein	[49]
*tet*(M)	F-ACAGAAAGCTTATTATATAACR-TGGCGTGTCTATGATGTTCAC	55	Tetracyclines	Ribosomal Protection Protein	[49]
*tet(O)*	F-ACGGARAGTTTATTGTATACCR-TGGCGTATCTATAATGTTGAC	60	Tetracyclines	Ribosomal Protection Protein	[49]
*tetA*(B)	F-TTGGTTAGGGGCAAGTTTTGR-GTAATGGGCCAATAACACCG	55	Tetracyclines	Efflux pump	[63]
*bla*_tem_	F-TTTCGTGTCGCCCTTATTCCR-CCGGCTCCAGATTTATCAGC	60	Penicillins	β-lactamase	[63]
*bla*_CTX-M_	F-ATGTGCAGYACCAGTAARGTKATGGCR-GGGTRAARTARGTSACCAGAAYSAGCGG	60	Penicillins	β-lactamase	[50]
*bla*_SHV_	F-CACTCAAGGATGTATTGTGR-TTAGCGTTGCCAGTGCTCG	58	Penicillins	β-lactamase	[64]
*mec*A	F-GGGATCATAGCGTCATTATTCR-AACGATTGTGACACGATAGCC	56	Penicillins	Penicillin-binding protein 2a	[50]
*aac*(6″)*-Ie-aph*(2″)	F-CCAAGAGCAATAAGGGCATACCR-CACACTATCATAACCATCACCG	55	Aminoglycosides	Bifunctional acetyltransferase phosphotransferase	[64]
*str*A	F-CTTGGTGATAACGGCAATTCR-CCAATCGCAGATAGAAGGC	65	Aminoglycosides	Phosphotransferase	[63]
*cml*A1	F-CACCAATCATGACCAAGR-GGCATCACTCGGCATGGACATG	60	Chloramphenicol	Efflux pump	[63]

### Statistical analyses

Results were analyzed using the SPSS software (SPSS Inc. Chicago, USA). The normality of the data, at each sampling point, was checked, and some of the bacterial groups showed non-normal distribution; therefore, differences between groups of infants were analyzed using non-parametric tests (Mann-Whitney *U* test or Kruskal-Wallis test). Pearson *χ* ^2^ test and logistic regression were used for assessing the occurrence of the different antibiotic resistance genes analyzed in both groups.

### Nucleotide sequence accession numbers

The raw sequences reported in this article have been deposited in the NCBI Short Read Archive (SRA) under accession number PRJNA362530.

## Results

### Impact of IAP on the intestinal microbiota composition of the newborn

Sequencing of the PCR products obtained by amplification of the V3-V4 region of the 16S rRNA gene from the samples analyzed yielded an average of ~60,000 filtered partial sequences per sample with an average length of 178 bp. A minimum of 47,000 sequences per sample were used for standardizing the microbiota measures. Rarefaction curves indicated that the sequencing depth was enough since the samples reached the plateau phase (Additional file 1: Figure S1). IAP was found to reduce alpha-diversity in comparison with no-IAP infants (Additional file 2: Figure S2).

**Fig. 1:**
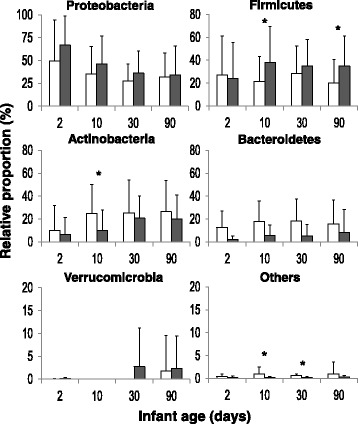
Relative proportions (%) of the main bacterial phyla present in the fecal samples obtained from both infant groups, newborns whose mothers received IAP (*black columns*) and those whose mothers dis not (white columns), at the different sampling points. *Asterisk* indicates statistically significant differences (*p* < 0.05) between both groups of infants

The microbiota of our full-term vaginally delivered infants was initially (2 days of age) dominated by *Proteobacteria* which represented a relative proportion of 67% in infants from mothers receiving IAP and of 50% in non-IAP-exposed infants (Fig. 1). The levels of this bacterial phylum decreased along time reaching levels of 46% in IAP-exposed and 35% in non-exposed infants at 10 days of age. These levels were further reduced to 36 and 27%, respectively, at 1 month of age and 34 and 32% at the age of 3 months. In spite of these general lower levels of *Proteobacteria* in non-IAP-exposed babies, compared with infants from IAP-receiving mothers, the differences did not reach statistical significance (*p* > 0.05), likely due to the high inter-individual variation. The level of *Firmicutes* in IAP infants increased from an initial 24% at 2 days of age to a 38% at 10 days, the levels remaining stable afterwards. In contrast, the levels of this microbial phylum did not show this increase and were maintained constant in the control group (infants not exposed to IAP), with only minor variations along time (Fig. 1). This different behavior of *Firmicutes* in IAP vs. control infants resulted in significantly higher levels (*p* < 0.05) of this phylum in the former group at 10 and 90 days of age. On the contrary to that observed for *Proteobacteria* and *Firmicutes*, the relative proportions of *Actinobacteria*, *Bacteroidetes*, and others were higher in control than in IAP-exposed infants (Fig. 1). These differences reached statistical significance at 10 days of age for *Actinobacteria*.

Analyses of the data at family level confirmed these differences, with some families showing statistically significant changes related to the use of IAP (Additional file 3: Table S1). Among these, it is important to underline the significantly lower levels of *Bifidobacteriaceae* and unclassified *Actinobacteria* (*p* < 0.05) found in IAP infants. On the contrary, the latter group of infants presented increased levels (*p* < 0.05) of *Prevotellaceae* at 2 and 90 days of age, *Rikenellaceae* at 2 days of age, *Clostridiaceae* at 10 days of age, and of *Campylobacteriaceae* and *Helicobacteriaceae* at the end of the study (3 months). Moreover, the levels of the group classified as Family S24–7 (*Muribaculaceae*), a *Bacteroidetes* group commonly found in feces from homoeothermic animals [39], but whose first cultured representative remained elusive till recently [40], were significantly increased in IAP infants all along the study.

### Impact of IAP on the intestinal production of SCFAs in the infant gut

**Fig. 2:**
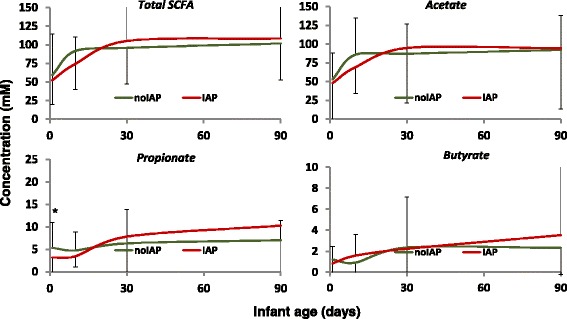
Concentration (mM) of acetate, propionate, and butyrate and total SCFAs in feces from infants whose mothers received IAP (IAP; *n* = 18) and those whose mothers did not (noIAP; *n* = 22)

The levels of the main fecal SCFAs (acetate, propionate, and butyrate) increased over time in both infant groups. Infants whose mothers received IAP showed a trend towards lower levels of propionate and acetate during the first days of life (Fig. 2). These differences were statistically significant (*p* < 0.05) for propionate at 2 days of life, when the levels of these SCFAs in infants not exposed to IAP almost doubled those in IAP infants (5.3 vs 3.1 mM, respectively). However, likely due to the high inter-individual variability observed in the levels of fecal SCFAs, no other statistically significant differences were obtained. This apparent delay in the production of acetate and propionate during the first days of life disappeared already at 30 days of age, when the levels of SCFAs in IAP infants were even slightly higher (*p* > 0.05) than in the control infants, and remained mostly unchanged during the rest of the study. When the SCFAs were calculated as relative proportions (percentage with regard to the total SCFAs level), no statistically significant differences were observed at any time point analyzed (Additional file 4: Figure S3).

### Effect of feeding habits on the impact of IAP upon the infant intestinal microbiota composition

To assess the potential influence of the infant feeding habits upon the observed effects of IAP on the establishing infant gut microbiota, we compared the data obtained for the exclusively breast-fed babies with those for formula-fed ones, both in mothers receiving and not receiving IAP. The results showed differences in the microbiota depending on the infant feeding habits as well as differential responses to IAP between both infant groups (Additional file 5: Table S2). Exclusively breast-fed infants not exposed to IAP showed higher levels of *Bacteroidetes* all along the study and lower early levels of *Proteobacteria* than unexposed formula-fed ones. Interestingly, regardless of IAP exposure, lower levels of *Actinobacteria* were observed in formula-fed newborns during early days life, but this trend changed afterwards with formula-fed infants showing higher levels after 1 month of age. Similarly, *Verrucomicrobia* levels were higher in the formula-fed group at the end of the study (90 days of age).

**Fig. 3:**
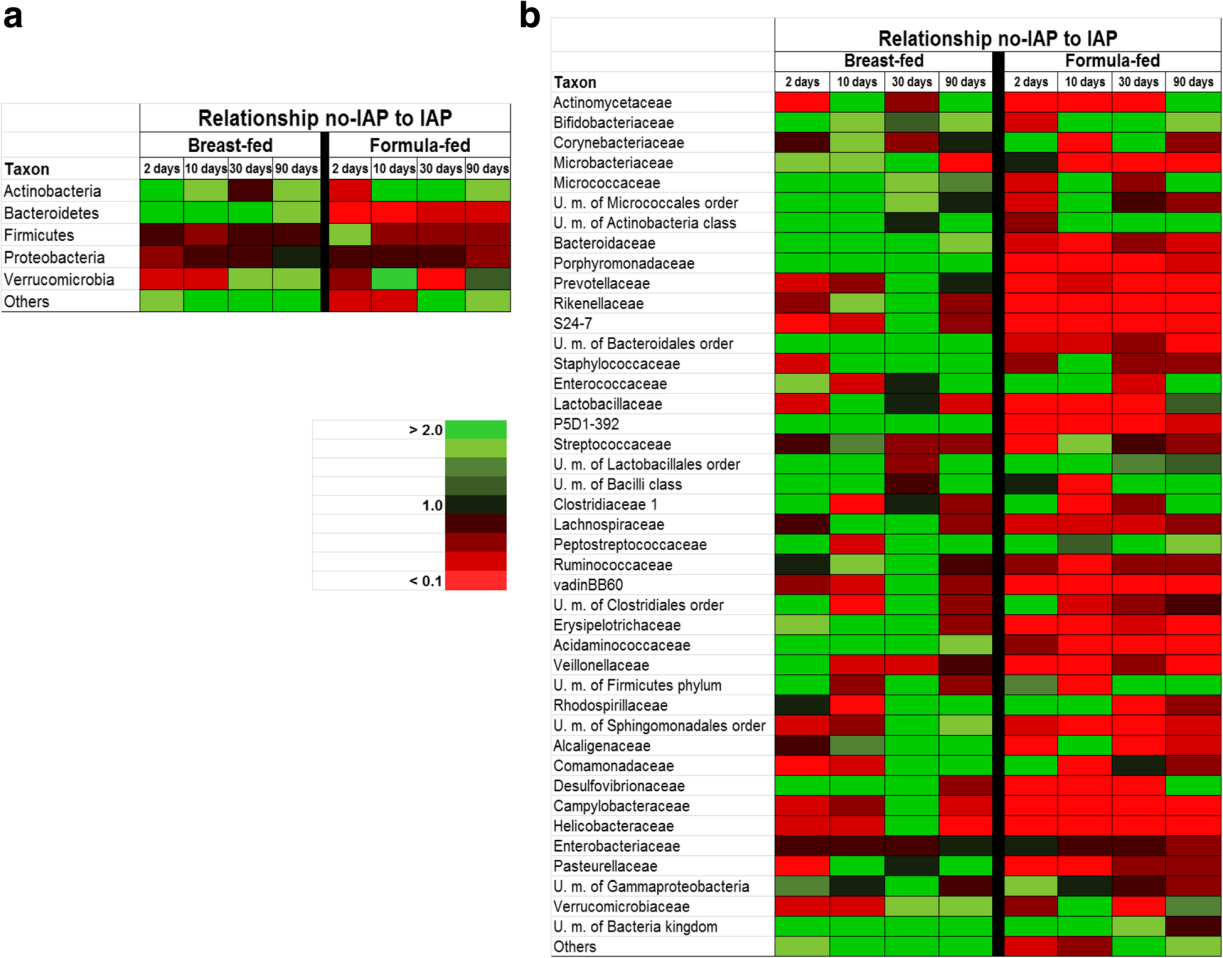
Ratio obtained for the different microbial phyla (**a**) and families (**b**) between no-IAP exposed infants (babies whose mothers did not receive IAP) and IAP-exposed ones in exclusively breast-fed infants (*n* = 29, 10 no-IAP and 11 IAP) and in formula-fed ones (*n* = 11, 2 no-IAP and 7 IAP). The ratio was calculated as relative proportion in no-IAP/relative proportion in IAP newborns at the different time points analyzed

Regarding the response to IAP, antibiotics exposure reduced the levels of *Actinobacteria* and increased those of *Firmicutes*, and to a lesser extent *Proteobacteria*, in both breast and formula-fed infants (Additional file 5: Table S2). In the case of *Bacteroidetes* the response to IAP was variable depending on the infant feeding pattern; thus whereas IAP reduced the levels of this phylum in exclusively breast-fed infants, the contrary was true in formula-fed ones. To further delineate this differential behavior, the relationship between non-IAP and IAP group was calculated for the different microorganisms in both breast-fed and formula-fed infants (Fig. 3). As expected from the composition data, the levels of *Proteobacteria* and *Firmicutes* were higher in IAP-exposed infants rendering a low ratio. This was true for both breast- and formula-fed infants. However, in the case of *Actinobacteria* and to a larger extent of *Bacteroidetes*, the impact of IAP seems to be different between breast-fed and formula-fed babies (Fig. 3). This is especially true for *Bacteroidetes* since all the main representative families of this phylum (*Bacteroidaceae*, *Prophyromonadaceae*, and *Prevotellaceae*) showed a lower ratio between non-IAP and IAP babies in the case of the formula-fed newborns (Fig. 3). Similar results were also obtained for the families *P5D1-392*, including unculturable microorganism from the *Lactobacillales* order, *Lachnospiraceae*, *Ruminococcaceae*, or *Acidaminococcaceae*, among others (Fig. 3).

### Impact of IAP on the carriage of antibiotic resistance genes

The assessment of the occurrence of different antibiotic resistance genes in the feces of 1-month-old infants by PCR with specific primer pairs showed variability among the analyzed genes (Table 2). Whilst, the genes *tet*M, *tet*O, *tet*A, *str*A, or *clm*A1 were not detected in any sample; others such as *BLA*-tem, *mec*A, or *CTX*-M resulted positive in more than 30% of the infants. Interestingly, the β-lactamase genes *BLA-tem*, *CTX-M*, and the gene acc6-aph2, conferring resistance to aminoglycosides, showed an increased occurrence in infants from IAP-receiving mothers than in non-antibiotic exposed ones (Table 2). However, these differences did not reach statistical significance (*p* > 0.05); although in the case of *BLA-tem*, a trend towards increased occurrence seems to be present (*χ* ^2^; 0.05 < *p* < 0.01), which is in agreement with the logistic regression analyses that also suggest IAP as a predictor of the occurrence of this gene (Odds ratio 4.3; *p* < 0.1).

**Table 2:** Occurrence (% of positive samples) of the different antibiotic resistance genes analyzed, in fecal samples from 1-month-old infants from mothers receiving IAP (*n* = 18) and those from mothers that did not receive intrapartum antibiotics (*n* = 22)

Group	Antibiotic resistance gene^a^					
	*tetW*	*BLA-tem*	*mecA*	*CTX-M*	*BLA-shv*	*acc6-aph2*
No-IAP	9.0%	61.9%	57.1%	33.3%	14.3%	23.8%
IAP	7.7%	82.3%	44.4%	52.9%	11.1%	38.9%

^a^None of the samples was positive for *tet*M, *tet*O, *tet*A, *str*A, or *cml*A1

## Discussion

During the last decade, we have started to understand the critical role of the early-life microbiota-host interaction as a determinant of later health [2, 41]. To this regard, the use of antibiotics may influence gut microbiota composition with consequences for later health [42]. Moreover, incomplete microbiota recovery after antibiotics administration, in both adults and infants, has been observed [43, 44]. These results point out to the need of defining and understanding the factors, such as IAP, which may determine the initial establishment of the gut microbiota.

In general, the composition of the gut microbiota in our study is similar to that reported by other authors in other full-term vaginally delivered baby cohorts [17, 32, 34, 44, 45]. As it is common in this typology of studies, a large inter-individual variability was observed, which hampers the statistical analyses of the results. Despite this large variability, our results demonstrate the impact of maternal IAP upon the composition of the gut microbiota in the infant during the first months of life. Similarly to other recent 16S rRNA gene-based studies, carried out in both preterm and full-term infants [17, 31–33], we observed a reduction on the levels of *Actinobacteria* and *Bacteroidetes* and an increase of *Proteobacteria* and *Firmicutes* in babies from mothers receiving IAP than in those not exposed to antibiotics. To note, infants in the IAP group showed reduced proportions of commensal microorganisms, such as the family *Bifidobacteriaceae*, but increased of potentially pathogenic microorganisms including *Campylobacteriaceae* or *Helicobacteriaceae*. Some of these differences were only present during a certain time, disappearing afterwards. For instance, infants whose mothers received IAP showed lower relative proportions of *Bifidobacteriaceae* at 10 days of age but not at the age of 1 month, which is in good agreement with the results obtained by Corvaglia and coworkers [46] by using qPCR for the genus *Bifidobacterium*. Moreover, our results indicate that some IAP-induced microbiota alterations remain for up to 3 months of age. To this regard, Mazzola and coworkers [33] recently reported similar differences to those obtained by us, persisting up to 1 month of age, which was the duration of their study. Similar to our results, Azad et al. [32] have also recently reported reduced levels of *Bacteroidetes* and increased of *Proteobacteria* in 3-month-old vaginally delivered infants whose mothers received IAP, these differences disappearing by the age of 1 year. Moreover, a similar pattern with increased colonization by potentially pathogenic enterobacteria has also been observed in preterm babies [17]. A question that remains open is the exact mechanism behind the observed effects. The spectrum of action of the antibiotic used, penicillin, may partially explain the observed results since it has a strong activity against gram-positive microorganisms and by reducing them would allow the overgrowth of gram-negatives such as Proteobacteria. However, this can be more complex and depend on the specific spectrum since *Firmicutes* (gram-positive) also show higher levels in IAP infants whilst the levels of other gram-negative microorganisms, such as those from the phyla *Bacteroidetes*, are reduced.

The information available on the impact of antibiotic exposure in SCFA production during early life is very scarce. Antibiotic use had been suggested as a potential cause for low fecal SCFA levels [47], and we recently reported lower levels of fecal SCFAs, especially of acetate, in preterm infants exposed to antibiotics [38]. In the same way, in the present study, we observed a trend towards a delayed increase of fecal acetate levels in infants from mothers receiving IAP in comparison with babies not exposed to antibiotics. Given the important roles that the SCFAs play in human physiology [48], this reduced acetate level during the first month of life could have potential effects on infant development and health.

Our results suggest a potential interaction between IAP administration and infant feeding habit. This is especially relevant for the phylum *Bacteroidetes* whose levels were much lower in formula-fed than in exclusively breast-fed infants. These differences seem to affect the response to IAP that differs between both groups of infants, giving an extra complexity to the understanding of the effects of early-life factors upon the microbiota. To this regard, Mazzola and coworkers [33] recently reported larger effects of IAP in the microbiota of exclusively breast-fed babies than in infants under mixed feeding. On the contrary, other authors found a potential protective role of breast-feeding against the IAP-induced microbiota changes in C-section-delivered babies [32]. Different methodological issues such as the limited number of newborns included in these studies, the several potentially confounding factors involved, as well as the challenge of defining infant-feeding regime other than exclusive breast-feeding, have difficulty in drawing firm conclusions. To this regard, our results point out to a differential response to IAP depending on infant feeding which adds more difficulty for reaching general conclusions or making recommendations. However, it would be important to define whether or not a protective role of breast-feeding is actually present since this would provide a rationale for additional efforts to encourage breast-feeding in those cases in which IAP has been used.

Another interesting issue related to the early microbiota composition that has remained largely unexplored is the carriage of antibiotic resistance genes. The increase of multidrug resistant microorganisms is an important concern of public health authorities worldwide. Given the potential role of the gut microbiota as reservoir for antibiotic resistance genes, the early microbiota establishment may also constitute a critical step from this perspective. Some studies have reported the presence of antibiotic resistance genes in the early life microbiota [49, 50]. Gosalbes and co-workers [50] demonstrated the presence of genes conferring resistance to β-lactam antibiotics and tetracycline in the meconium of more than half of the newborns. Moreover, the results of these authors suggest the vertical mother-infant transmission of antibiotic resistance genes and underline the potential role of the infant microbiome as a reservoir of resistance genes [50]. Gibson et al. [51] recently reported the enrichment of specific antibiotic genes following antibiotic treatments in preterm infants. However, although IAP is a fairly common practice and the most frequent cause of antibiotics exposure to antibiotics in neonates, no study has addressed, until now, the possible impact of this practice on the carriage of antibiotic resistance genes by the infant microbiota. In the present study, we have focused on genes previously reported to be relatively common in infants [49–51] and observed an increased number of infants harboring genes, such as *BLA-tem*, conferring resistance to the antibiotics used in IAP (β-lactam) in the group of newborns whose mothers received IAP. On the contrary, no differences between infants from others receiving and not receiving IAP were observed for genes conferring resistance to antibiotics that are not used in IAP, such as tetracycline. Although the results did not reach statistical significance, likely due to the limited sample size, the observed trend towards an increased carriage of *BLA-tem* following IAP, with an additional 20% of infants harboring the gene, deserves further attention and confirmation in larger studies.

Our results, together with those recently reported by other authors, indicate an effect of IAP on the establishing early microbiota. Interestingly, this effect seems to remain for at least the first months of life which represent a very critical time for the correct development of the microbiota-induced host homeostasis [2, 41]. The use of IAP is not a clear-cut decision [52] and is usually recommended in cases in which prenatal screening demonstrates colonization by group B streptococci [53]. Unfortunately, IAP is often also administered in cases in which there is not a clear benefit [54, 55] and there is a high rate of administration of inadequate IAP [56]. In general, inappropriate antibiotic use is frequent in the pediatric population, which is a matter of concern [57]. Moreover, it is becoming increasingly evident that antibiotic-induced perturbation in the early microbiota may have profound consequences for later health. Different animal studies have demonstrated that the alteration of the early microbiota with antibiotics increases the risk for autoimmune and metabolic diseases, also affecting behavior [24, 25, 58–60]. Actually, repeated exposure to β-lactam antibiotics during infancy has been related with an increased weight in later life [61], and the intestinal microbiome of infants has been repeatedly reported to be affected by antibiotic use [21, 62]. All these data point out at the urgent need to rationalize the use of antibiotics and to develop intervention strategies aimed at minimizing the impact of early-life antibiotics exposure on the intestinal microbiota development process.

## Conclusions

This study underlines the effect of maternal IAP on the process of establishment of the intestinal microbiota in the newborn. The initial stages of development may represent the window of opportunity for intestinal microbiota modulation, either towards the establishment of a healthy microbial profile or to an aberrant profile. Thus, it is essential to understand how different perinatal factors, such as the use of IAP, may affect the gut microbiota development in the infant and to develop, if needed, treatments to minimize the impact on the microbiota development of any early-life intervention.

## Additional files

Additional file 1: Figure S1. Rarefaction curves generated for the 16S rRNA sequences obtained from the samples using Chao 1 index (A) and Shannon index (B) (PPTX 192 kb)

Additional file 2: Figure S2. Box plot of mean alpha diversity obtained by combining the data on infants born from mothers receiving IAP (*n* = 18) or those whose mothers did not receive IAP (*n* = 22) at 2, 10, 30, and 90 days of age. (PPTX 57 kb)

Additional file 3: Table S1. Levels (relative frequencies; %) of the bacterial families showing differences, in at least one time point analyzed, between infants from mothers receiving IAP and those whose mothers did not receive it. (DOCX 17 kb)

Additional file 4: Figure S3. Relative proportions (%) of the main SCFAs (acetate, propionate, and butyrate) in feces from infants whose mothers received intrapartum antimicrobial prophylaxis (IAP) and those whose mothers did not (no IAP). (PPTX 91 kb)

Additional file 5: Table S2. Relative proportion (%; mean ± sd) of the five main bacterial phyla in the samples from breast and formula-fed infants either exposed of not to IAP. Asterisk denotes statistically significant differences (*p* < 0.05) between non-IAP- and IAP-exposed infants within the same group. ^$^Denotes statistically significant differences (*p* < 0.05) between breast-fed and formula-fed infants within the same IAP exposition group. (DOCX 17 kb)
