# Supplementary material for: Impact of intrapartum antimicrobial prophylaxis upon the intestinal microbiota and the prevalence of antibiotic resistance genes in vaginally delivered full-term neonates
Source: Microbiome. 2017 Aug 8;5:93. doi: 10.1186/s40168-017-0313-3 (PMC5549288; doi:10.1186/s40168-017-0313-3)
Supplement: Supplementary file 1 — Rarefaction curves generated for the 16S rRNA sequences obtained from the samples using Chao 1 index (A) and Shannon index (B) (PPTX 192 kb) [file 40168_2017_313_MOESM1_ESM.pptx]

## Slide 1
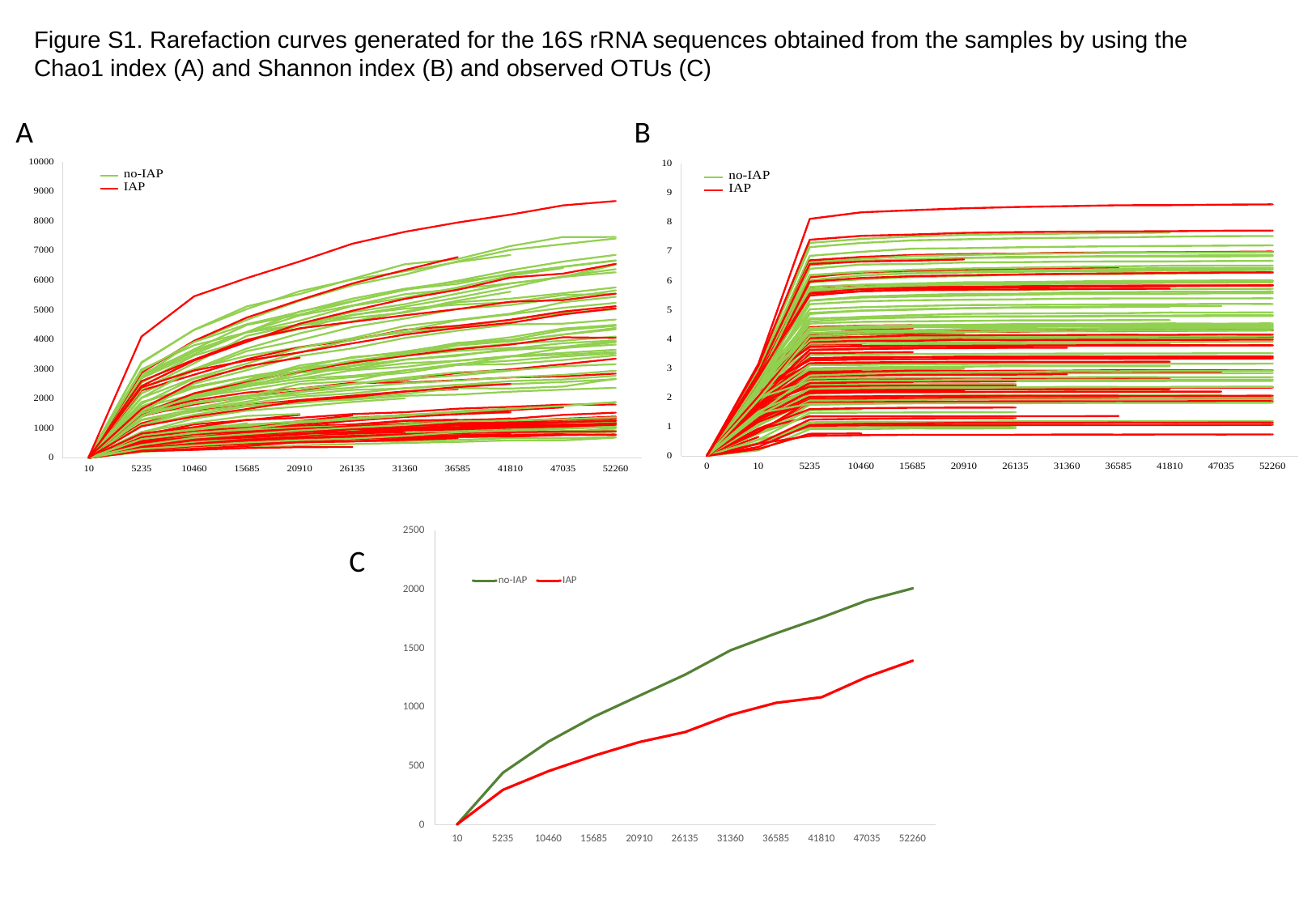

Figure S1. Rarefaction curves generated for the 16S rRNA sequences obtained from the samples by using the Chao1 index (A) and Shannon index (B) and observed OTUs (C)
A
B
C
