# Supplementary material for: Impact of intrapartum antimicrobial prophylaxis upon the intestinal microbiota and the prevalence of antibiotic resistance genes in vaginally delivered full-term neonates
Source: Microbiome. 2017 Aug 8;5:93. doi: 10.1186/s40168-017-0313-3 (PMC5549288; doi:10.1186/s40168-017-0313-3)
Supplement: Supplementary file 2 — Box plot of mean alpha diversity obtained by combining the data on infants born from mothers receiving IAP (n = 18) or those whose mothers did not receive IAP (n = 22) at 2, 10, 30, and 90 days of age. (PPTX 57 kb) [file 40168_2017_313_MOESM2_ESM.pptx]

## Slide 1
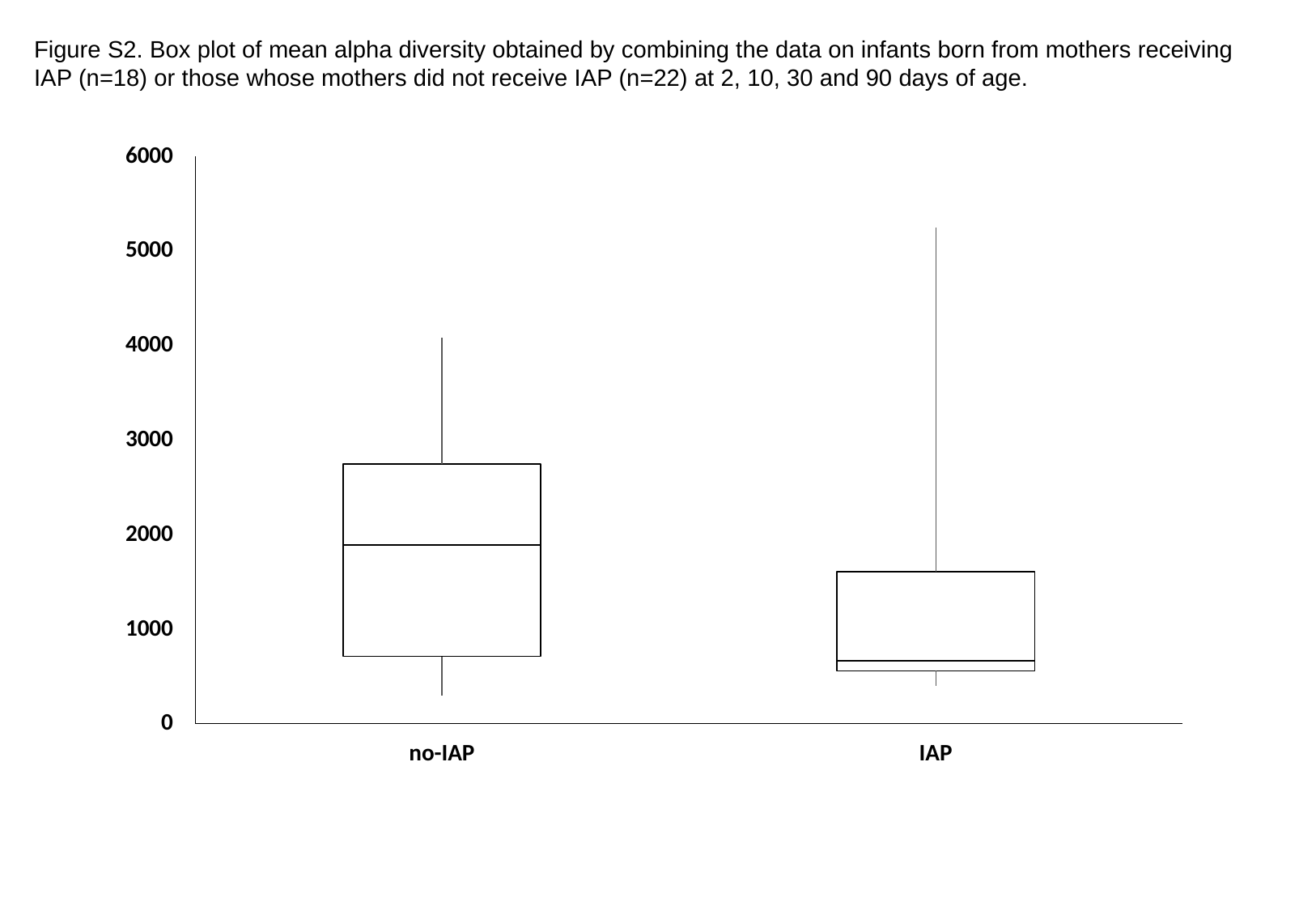

Figure S2. Box plot of mean alpha diversity obtained by combining the data on infants born from mothers receiving IAP (n=18) or those whose mothers did not receive IAP (n=22) at 2, 10, 30 and 90 days of age.
