# Supplementary material for: Impact of intrapartum antimicrobial prophylaxis upon the intestinal microbiota and the prevalence of antibiotic resistance genes in vaginally delivered full-term neonates
Source: Microbiome. 2017 Aug 8;5:93. doi: 10.1186/s40168-017-0313-3 (PMC5549288; doi:10.1186/s40168-017-0313-3)
Supplement: Supplementary file 3 — Levels (relative frequencies; %) of the bacterial families showing differences, in at least one time point analyzed, between infants from mothers receiving IAP and those whose mothers did not receive it. (DOCX 17 kb) [file 40168_2017_313_MOESM3_ESM.docx]

**Table S1.** Levels (relative frequencies; %) of the bacterial families showing differences, in at least one time point analyzed, between infants from mothers receiving IAP and those whose mothers did not receive it.

| **Bacterial family** | **Infant age  (days)** | **No-IAP  (mean ± sd)** | **IAP  (mean ± sd)** | ***P* value** |
| --- | --- | --- | --- | --- |
| *Bifidobacteriaceae* | 2 10 30 90 | 8.69 ± 21.24 22.14 ± 23.19 22.58 ± 29.13 26.24 ± 25.96 | 6.36 ± 14.32 8.87 ± 17.36 20.31 ± 19.41 19.11 ± 20.27 | 0.460 ***0.011*** 0.996 0.257 |
| *Campylobacteriaceae* | 2 10 30 90 | 0.01 ± 0.03 0.01 ± 0.02  0.06 ± 0.20 0.00 ± 0.01 | 0.03 ± 0.04 0.03 ± 0.06 0.03 ± 0.06 0.03 ± 0.05 | 0.174 0.209 0.657 ***0.039*** |
| *Clostridaceae* | 2 10 30 90 | 6.64 ± 19.79 0.59 ± 2.15 3.22 ± 9.26 2.50 ± 5.98 | 0.29 ± 0.96 16.04 ± 26.62 2.35 ± 6.61 5.80 ± 13.84 | 0.191 ***0.027*** 0.925 0.407 |
| *Helicobacteraceae* | 2 10 30 90 | 0.11 ± 0.26 0.11 ± 0.42 0.18 ± 1.42 0.03 ± 0.08 | 0.80 ± 1.28 0.76 ± 1.47 0.78 ± 1.52 0.87 ± 1.71 | 0.078 0.089 0.666 ***0.039*** |
| *Prevotellaceae* | 2 10 30 90 | 0.02 ± 0.03 0.02 ± 0.03 0.12 ± 0.34 0.03 ± 0.05 | 0.12 ± 0.20 0.07 ± 0.13 0.07 ± 0.12 0.08 ± 0.11 | ***0.040*** 0.338 0.299 ***0.028*** |
| *Rikenellaceae* | 2 10 30 90 | 0.12 ± 0.29 0.25 ± 0.97 0.58 ± 1.48 0.03 ± 0.04 | 0.28 ± 0.35 0.24 ± 0.45 0.22 ± 0.37 0.24 ± 0.40 | ***0.017*** 0.190 0.443 0.080 |
| *S24_7* | 2 10 30 90 | 0.04 ± 0.07 0.04 ± 0.11 0.22 ± 0.37 0.03 ± 0.05 | 0.43 ± 0.79 0.24 ± 0.43 0.46 ± 1.47 0.25 ± 0.43 | ***0.004 0.045 0.039 0.024*** |
| Unclassified Actinobacteria | 2 10 30 90 | 0.18 ± 0.46 0.91 ± 3.41 0.15 ± 0.15 0.38 ± 0.51 | 0.04 ± 0.08 0.01 ± 0.02 0.11 ± 0.20 0.11 ± 0.25 | ***0.026 0.001*** 0.084 ***0.003*** |
| Unclassified Bacteria | 2 10 30 90 | 0.32 ± 0.49 0.89 ± 1.40 0.56 ± 1.21 0.85 ± 2.06 | 0.12 ± 0.20 0.14 ± 0.24 0.12 ± 0.20 0.21 ± 0.28 | 0.106 ***0.022 0.037*** 0.244 |
