# Supplementary material for: Impact of intrapartum antimicrobial prophylaxis upon the intestinal microbiota and the prevalence of antibiotic resistance genes in vaginally delivered full-term neonates
Source: Microbiome. 2017 Aug 8;5:93. doi: 10.1186/s40168-017-0313-3 (PMC5549288; doi:10.1186/s40168-017-0313-3)
Supplement: Supplementary file 4 — Relative proportions (%) of the main SCFAs (acetate, propionate, and butyrate) in feces from infants whose mothers received intrapartum antimicrobial prophylaxis (IAP) and those whose mothers did not (no IAP). (PPTX 91 kb) [file 40168_2017_313_MOESM4_ESM.pptx]

## Slide 1
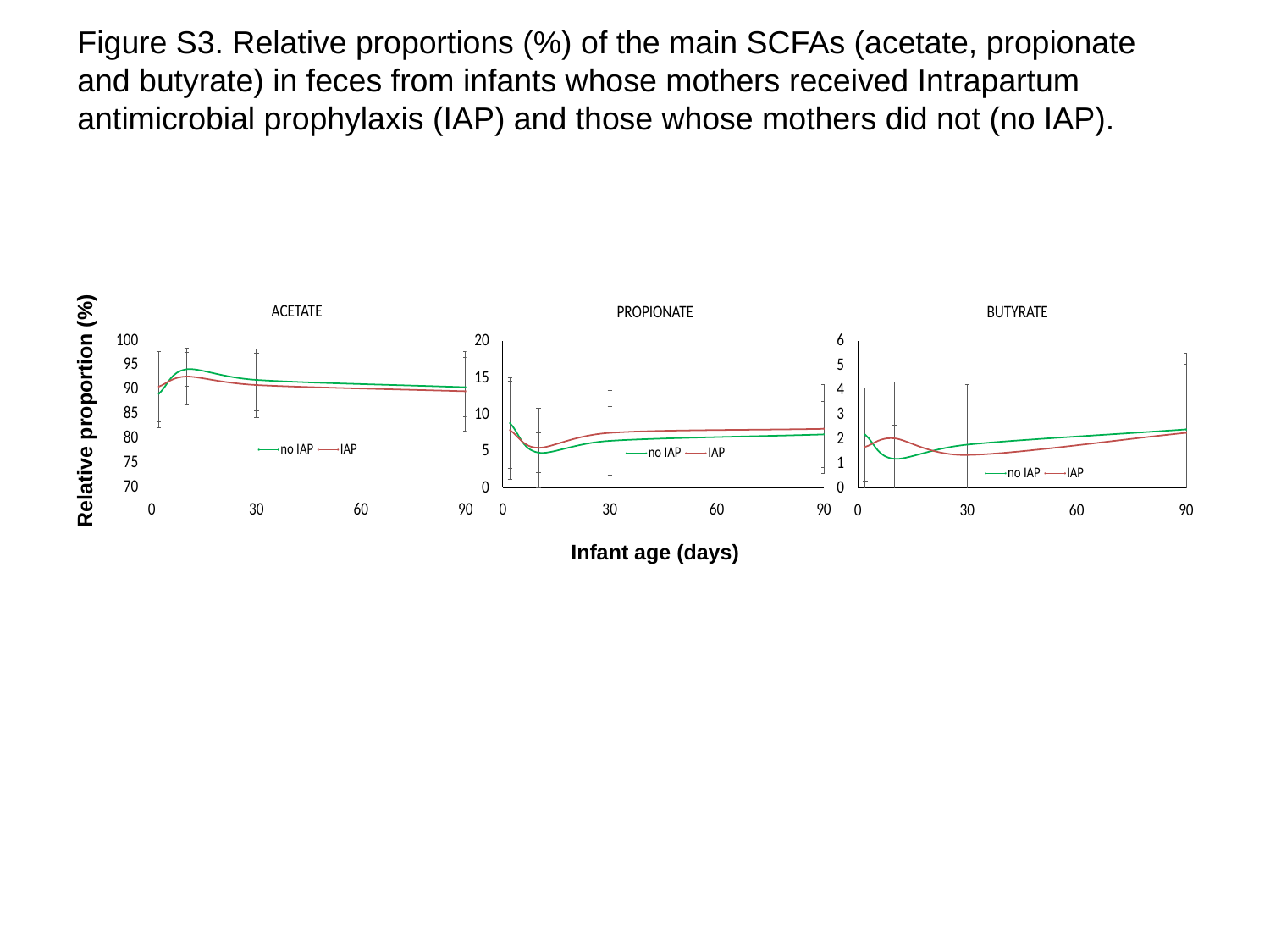

Figure S3. Relative proportions (%) of the main SCFAs (acetate, propionate and butyrate) in feces from infants whose mothers received Intrapartum antimicrobial prophylaxis (IAP) and those whose mothers did not (no IAP).
Relative proportion (%)
Infant age (days)
