# Supplementary material for: Impact of intrapartum antimicrobial prophylaxis upon the intestinal microbiota and the prevalence of antibiotic resistance genes in vaginally delivered full-term neonates
Source: Microbiome. 2017 Aug 8;5:93. doi: 10.1186/s40168-017-0313-3 (PMC5549288; doi:10.1186/s40168-017-0313-3)
Supplement: Supplementary file 5 — Relative proportion (%; mean ± sd) of the five main bacterial phyla in the samples from breast and formula-fed infants either exposed of not to IAP. Asterisk denotes statistically significant differences (p < 0.05) between non-IAP- and IAP-exposed infants within the same group. $Denotes statistically significant differences (p < 0.05) between breast-fed and formula-fed infants within the same IAP exposition group. (DOCX 17 kb) [file 40168_2017_313_MOESM5_ESM.docx]

**Table S2.** Relative proportion (%; mean ± sd) of the five main bacterial phyla in the samples from breast and formula-fed infants either exposed of not to IAP. * Denotes statistically significant differences (p<0.05) between no-IAP and IAP exposed infants within the same group. ^$^ Denotes statistically significant differences (p<0.05) between breast-fed and formula-fed infants within the same IAP exposition group.

| **Phylum** | **Age** | **Breast-fed** | |  | **Formula-fed** | |
| --- | --- | --- | --- | --- | --- | --- |
|  |  | *No-IAP* | *IAP* |  | *No-IAP* | *IAP* |
| **Actinobacteria** | 2 days | 11.87 ± 23.66 | 5.89 ± 10.57 |  | 2.18 ± 1.29 | 8.04 ± 20.16 |
|  | 10 days | 22.66 ± 25.15* | 12.30 ± 21.57* |  | 34.01 ± 28.26* | 6.06 ± 9.68* |
|  | 30 days | 17.96 ± 10.43^$^ | 19.79 ± 19.84 |  | 58.68 ± 43.09^$^ | 22.27 ± 20.36 |
|  | 90 days | 21.49 ± 22.36^$^ | 13.23 ± 15.75 |  | 61.48 ± 21.89^$^ | 32.02 ±25.27 |
| **Bacteroidetes** | 2 days | 15.69 ± 30.76^$^ | 3.08 ± 3.30 |  | 0.11 ± 0.19^$^ | 1.08 ± 1.10 |
|  | 10 days | 22.03 ± 30.19* | 1.66 ± 2.76*^$^ |  | 0.32 ± 0.36* | 11.8 ± 12.36*^$^ |
|  | 30 days | 21.79 ± 27.71 | 4.33 ± 12.67 |  | 1.62 ± 1.74 | 6.47 ± 5.44 |
|  | 90 days | 19.53 ± 28.21 | 11.72 ± 24.37 |  | 0.34 ± 0.30* | 2.50 ± 2.53* |
| **Firmicutes** | 2 days | 27.88 ± 33.46 | 30.10 ± 35.67 |  | 24.21 ± 42.34 | 14.36 ± 23.00 |
|  | 10 days | 21.65 ± 23.10* | 45.29 ± 35.26* |  | 19.03 ± 20.76 | 26.75 ± 19.56 |
|  | 30 days | 32.24 ± 24.37 | 40.53 ± 22.92 |  | 11.31 ± 16.85 | 26.47 ± 22.36 |
|  | 90 days | 22.23 ± 22.60* | 40.41 ± 24.76* |  | 10.73 ± 8.19 | 24.80 ± 27.68 |
| **Proteobacteria** | 2 days | 44.09 ± 44.50 | 60.66 ± 38.38 |  | 73.28 ± 43.69 | 76.37 ± 37.78 |
|  | 10 days | 32.56 ± 27.04 | 40.54 ± 34.20 |  | 46.51 ± 44.54 | 55.23 ± 23.87 |
|  | 30 days | 27.29 ± 16.60 | 35.13 ± 23.91 |  | 28.29 ± 30.51 | 37.67 ± 26.54 |
|  | 90 days | 35.56 ± 26.83 | 34.35 ± 31.34 |  | 18.56 ± 18.60 | 33.75 ± 36.12 |
| **Verrucomicrobia** | 2 days | 0.04 ± 0.11* | 0.16 ± 0.41* |  | 0.01 ± 0.02 | 0.01 ± 0.03 |
|  | 10 days | 0.01 ± 0.01 | 0.01 ± 0.02 |  | 0.01 ± 0.14 | 0.00 ± 0.01 |
|  | 30 days | 0.01 ± 0.03 | 0.01 ± 0.01 |  | 0.00 ± 0.00 | 7.04 ± 18.41 |
|  | 90 days | 0.01 ± 0.01^$^ | 0.00 ± 0.01^$^ |  | 8.72 ± 17.45^$^ | 6.68 ± 11.16^$^ |
